# Attention-Guided Disentangled Feature Aggregation for Video Object Detection

**DOI:** 10.3390/s22218583

**Published:** 2022-11-07

**Authors:** Shishir Muralidhara, Khurram Azeem Hashmi, Alain Pagani, Marcus Liwicki, Didier Stricker, Muhammad Zeshan Afzal

**Affiliations:** 1 Department of Computer Science, Technical University of Kaiserslautern, 67663 Kaiserslautern, Germany; 2 Mindgarage, Technical University of Kaiserslautern, 67663 Kaiserslautern, Germany; 3 German Research Institute for Artificial Intelligence (DFKI), 67663 Kaiserslautern, Germany; 4 Department of Computer Science, Luleå University of Technology, 971 87 Luleå, Sweden

**Keywords:** object detection, video object detection, attention, computer vision, deep learning

## Abstract

Object detection is a computer vision task that involves localisation and classification of objects in an image. Video data implicitly introduces several challenges, such as blur, occlusion and defocus, making video object detection more challenging in comparison to still image object detection, which is performed on individual and independent images. This paper tackles these challenges by proposing an attention-heavy framework for video object detection that aggregates the disentangled features extracted from individual frames. The proposed framework is a two-stage object detector based on the Faster R-CNN architecture. The disentanglement head integrates scale, spatial and task-aware attention and applies it to the features extracted by the backbone network across all the frames. Subsequently, the aggregation head incorporates temporal attention and improves detection in the target frame by aggregating the features of the support frames. These include the features extracted from the disentanglement network along with the temporal features. We evaluate the proposed framework using the ImageNet VID dataset and achieve a mean Average Precision (mAP) of 49.8 and 52.5 using the backbones of ResNet-50 and ResNet-101, respectively. The improvement in performance over the individual baseline methods validates the efficacy of the proposed approach.

## 1. Introduction

Computer Vision is an application of Artificial Intelligence (AI) focused on implementing human-like cognition and visual processing in computer systems. It has extensive applications, including autonomous driving [1], surveillance systems [2], agriculture [3] and healthcare [4]. Computer vision consists of tasks such as classification, where an image is classified into one of two or more classes; segmentation, where regions of interest are extracted from an image; tracking, where objects of interest are tracked across a video. Object detection is another such computer vision task that localizes and identifies the objects in an image, and this is achieved by predicting the coordinates of the object and subsequently classifying the object. Object detection methods can be categorised into two types, namely, one-stage object [5,6,7] and two-stage object detection [8,9,10]. In the case of one-stage object detection, both the bounding box prediction and the classification is carried out in a single stage without using pre-generated region proposals. Instead of proposals, one-stage detectors divide the image into grids of equal size, and each grid is used for the detection and localisation of the object it contains. On the contrary, a two-stage object detector generates the region proposals in the first stage. Proposals are regions in the image where the object might be present. In the next stage, the objects in the proposals are classified. Two-stage detectors have higher localisation and object recognition accuracy, whereas one-stage detectors achieve higher inference speeds [11].

**Figure 1:**
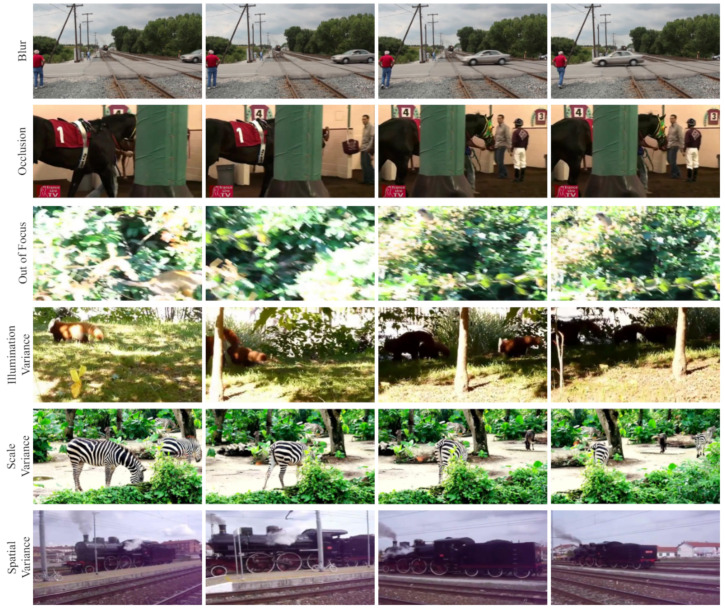
Illustration of challenges in video object detection. Unlike object detection in still images, objects suffer from appearance deterioration in videos caused by several challenges.

The simple application of still-image object detectors is sub-optimal in challenging environments [12,13]. Furthermore, applying image object detection algorithms to video data would process it as a sequence of unrelated individual images, and this approach would result in losing the temporal information present across the frames. The fundamental obstacle in Video Object Detection (VOD) is the appearance degradation of objects [14], which is caused by several challenging scenarios, as illustrated in Figure 1 and explained below:

- Motion Blur: Occurs due to the rapid or sudden movement of the objects, resulting in the object being blurry and losing its characteristics.
- Defocus: Occurs when the camera is not able to focus on the object in motion or when the imaging system itself is being moved. This results in unclear and out-of-focus frames.
- Occlusion: Occurs when the object is hidden behind other objects or elements in the environment. Occlusion results in a significant loss of information.
- Illumination Variance: Variation in the intensity of light can cause the object to have significantly different characteristics, such as losing colour information under a shadow.
- Scale Variance: The perceived size of the object changes as it moves towards or away from the camera and also when the camera zooms or moves with respect to the object.
- Spatial Variance: When the camera angle changes, it introduces different viewpoints, rotations or locations of the object, which may result in significantly different characteristics at different viewpoints.

The challenges of blurriness, camera defocus, occlusion and illumination variance can be overcome by leveraging information from the adjacent frames. Therefore, one of the keys to efficient video object detection is effectively leveraging the temporal information across video frames. Recent works have devised various feature aggregation methods [15,16,17] that leverage the attention mechanisms to aggregate features from video frames. Despite the remarkable improvement, the aggregation schemes in these approaches are naive and only operate on the semantic similarities among video frames. This yields sub-optimal performance due to the involved high intra-class similarity caused by spatial, scale and task in the video frames. Due to the fast motion in videos, objects in neighbouring frames can be of distinctive scales. Furthermore, since the object detection problem deals with multiple tasks, i.e., localisation and classification, we argue that learning task-specific features will produce better results. Similarly, due to high spatial variance among video frames, prior to aggregation learning, spatial-aware features will refine the target frame representation.

To validate this hypothesis, inspired by the unified attention head introduced in [18], we disentangle the feature representation first by learning three different feature representations for each video frame. Following [18], we name them scale-aware, spatial-aware and task-aware features. The attention head incorporates and integrates scale, spatial and task-aware attention, which assists in overcoming challenges of scale and spatial variance. We observe a consistent and significant rise in performance when these enhanced representations from support frames are aggregated with temporal attentional feature aggregation.

The use of attention also alleviates the challenge of processing large amounts of video data, as it focuses only on the relevant parts of the image.

To summarize, the primary contributions of this paper are as follows:

- We highlight an important problem that naive attention-based feature aggregation is sub-optimal for video object detection. As a remedy, we follow the spirits of [18] in still images and propose to disentangle the representation first through refined spatial, scale and task-specific feature learning, which compliments inter-frame feature aggregation for video object detection.
- The proposed approach can be effortlessly integrated into any video object detection method to improve performance.
- Upon integrating our disentanglement head prior to feature aggregation in recent video object detection methods, we observe consistent and significant performance improvements. When our module is adopted into the recent state-of-the-art method TROI [16], we achieve a new state-of-the-art mAP of 80.3% on the ResNet-50 backbone network.

The rest of the paper is organized as follows: In Section 2, we discuss the background, survey and categorize deep-learning-based approaches to video object detection. In Section 3, we present the proposed attention-based framework for video object detection and discuss each framework component in detail. Section 4 discusses the data and metrics used in the evaluation. The results and performance of the model are presented, discussed and visualised in Section 5. In Section 6, we conclude with a summary of our work.

## 2. Background and Related Works

**Figure 2:**
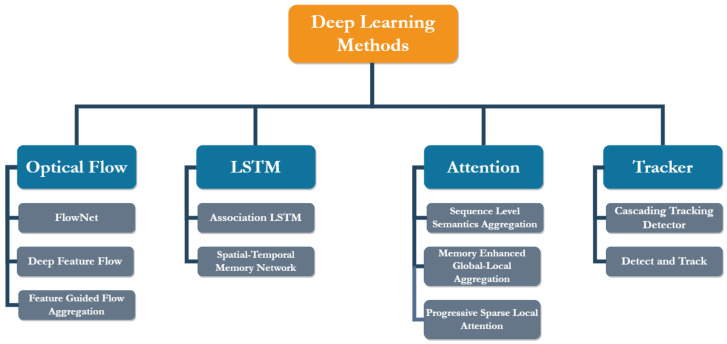
Overview of deep learning-based video object detection methods. The surveyed works have been categorized under the corresponding approaches.

Initial approaches to video object detection used handcrafted features, these included methods such as a histogram of oriented gradients (HOG) [19], scale-invariant feature transform (SIFT) [20], speed-up robust features (SURF) [21] and binary robust independent elementary features (BRIEF) [22]. The features used in these approaches have to be manually selected; therefore, the detector’s efficiency is highly dependent on the person’s expertise. Furthermore, as the number of classes increases, this process becomes cumbersome [23]. Addressing this shortcoming in traditional approaches, deep learning-based methods present end-to-end learning [24], where the network is presented with the data and their corresponding annotations, and during training, it automatically learns the underlying patterns and important features for each class. In this section, we survey deep learning-based methods for video object detection, and we categorize these methods into flow-based, context-based, attention-based and tracking-based methods, as shown in Figure 2.

### 2.1. Optical Flow-Based Methods

Optical flow algorithms detect object motion by assigning a velocity vector to each pixel [25], and when the light flow field of a pixel associated with an object changes with respect to a stationary object next to it, the object motion is registered. FlowNet [26], proposed by Dosovitskiy et al., extends this idea to deep learning, making it an end-to-end learning problem to predict optical flow.

Optical flow methods use the strong features from a key frame to strengthen the features in weaker frames. The challenge of selecting the key frame was addressed by Deep Feature Flow [27] (DFF). In DFF, a convolutional subnetwork of ResNet-101 is used to extract features from the sparse key frames. The features of non-key frames were extracted by propagating the feature maps of the key frame using a flow field. As it avoids feature extraction on each non-key frame, it reduces the computation time and accelerates object detection.

The Flow-guided Feature Aggregation [28] (FGFA) algorithm extends DFF, and the convolutional sub-network is applied to all the frames, i.e., every frame is treated as a key frame. For the current frame and a neighbour frame, a flow field is estimated, and the feature maps of the neighbour frame are warped onto the current frame according to the flow. The warped feature maps and the feature maps extracted from nearby frames are aggregated. The detection network utilizes the aggregated feature maps to perform detection on the current frame. FGFA achieves higher accuracy but at the cost of higher computation.

### 2.2. LSTM-Based Methods

Optical flow methods leverage the temporal context between only two frames and do not completely utilize the context present in the entire video data; an efficient object detector must be capable of utilizing the full contextual information across all frames. A convolutional LSTM [29] is a type of recurrent neural network that can be used to learn long-term spatio-temporal information through gates that extract and propagate the features.

Lu et al. [30] propose Association LSTM, a method that incorporates the association of objects between frames. Association LSTM consists of two modules; Single Shot Multibox Detector [5] (SSD), an object detection network, and convolutional LSTM. The SSD performs object detection on each frame, and then the features of the object are extracted and stacked. The stacked features are given as the input to the LSTM for processing each frame. For the output of the LSTM on adjacent frames, an association error is calculated and optimizing this loss maintains the temporal information of the object across the entire video.

A shortcoming in association LSTM is that the temporal context is limited to adjacent frames and, therefore, it uses only short-term motion information. This lack of long-term motion information was addressed by spatial-temporal memory network [31] (STMN). STMN receives the feature maps of the current frame and spatial-temporal memory with the information of all the previous frames. Subsequently, the spatial-temporal memory is updated with the current frame. It consists of two STMNs for bidirectional feature aggregation of both previous and future frames, and thereby the STMN encodes long-term information.

### 2.3. Attention-Based Methods

Another challenge with video data is the amount of information to be processed, which requires extensive computation. Unlike the previously discussed methods, attention-based methods aim to reduce the amount of computation by focusing only on certain parts of the data while ignoring the rest [32]. In the work, we adopt an attention-based method for video object detection. Sequence Level Semantics Aggregation [15] (SELSA) is one such method that extracts proposals from different frames and then computes the semantic similarity across the frames. Based on the similarities, the features from other frames are aggregated for robust detection. Performing aggregation on the proposal level instead of the feature map makes it more robust.

Memory-Enhanced Global–Local Aggregation [33] (MEGA) is modelled by how human perceive objects in a video—through global semantic information and local localisation information. MEGA consists of the global–local aggregation or the base model, which exploits the global features to enhance the local features by integrating global information into the local frames. The proposed Long-Range Memory strengthens the base model to use both global and local features in detection.

The Progressive Sparse Local Attention [34] (PSLA) module replaces the optical flow algorithm in establishing spatial correspondence between feature maps and propagating the features between frames. It performs feature extraction on the sparse key frames and then utilizes it for the non-key frames. PSLA proposes two methods, dense feature transformation (DenseFT), for propagating semantic information from the nearest key frame to the non-key frames. The recursive feature update (RFU) maintains long-term temporal information by updating the temporal feature at the key frames.

### 2.4. Tracking-Based Methods

The tracking algorithms process spatial and temporal information that can be leveraged for improving object detection. In contrast to optical flow methods that predict the trajectory of an object using a flow field, tracking-based methods are more accurate and have a longer trajectory. The cascading tracking detector [35] (CaTDet) consists of a detector and tracker, and the latter is used for reducing the computations. The detector predicts the target area in each frame of the video. The object’s position in the next frame is predicted by tracking the boxes of high confidence in the current frame using the tracker. For each frame, the output from the detector and the tracker are combined and inputted into the refinement network to obtain the calibrated information.

The Detect and Track [36] (D&T) approach integrates the detection and tracking modules into the same framework built upon the R-FCN [37]. D&T takes two input frames and computes the feature maps that are shared for both detection and tracking. The proposals are generated by the RPN, and RoI pooling is finally utilised in the final detection. The D&T approach overcomes the shortcomings in CaTDet of detecting occluded objects or predicting new objects in subsequent frames, and as a result, also reduces the computation.

### 2.5. Attention in Image Object Detection

Inspired by the human visual system, attention mechanisms are employed in computer vision tasks to emphasize the most important regions of an image while disregarding the other regions. Attention can be regarded as a dynamic mechanism that assesses the importance of the features and adapts the weights accordingly [32]. This section delves deeper into the use of attention mechanisms for object detection. Li et al. propose an adaptive attention mechanism [38] that integrates three adaptive attention units, namely, channel, spatial and domain. The channel attention unit extends the Squeeze-and-Excitation [39] structure by considering global max pooling in addition to global average pooling. Ying et al [40]. apply a multi-attention model comprising spatial, pixel and channel attention modules for object detection in aerial images. The pixel attention fuses local and global information at the pixel level, increasing the receptive field.

Carion et al. [41] introduce the Detection Transformer (DETR) architecture combining CNNs and transformer encoder–decoder. Through the use of transformers, DETR eliminates the need for methods such as non-maximum suppression and anchor generation, making it a truly end-to-end object detector. Addressing the limitations in DETR of slow convergence and poor performance in detecting small objects, Zhu et al. [42] propose the Deformable DETR. This is achieved through the proposed multi-scale deformable attention module that replaces the transformer attention modules for processing feature maps found in DETR. Dai et al. propose Dynamic DETR [43] for object detection using dynamic attention with transformers. Dynamic DETR implements attention in both the encoder and decoder, which overcomes the limitation of small feature resolution and training convergence in transformers.

Wang et al. tackle the challenge of scale variance by using spatial attention to refine multi-scale features [44]. The authors propose a Receptive Field Expansion Block (RFEB), which increases the receptive field size, and the features generated pass through the spatial refinement module to repair the spatial details of multi-scale objects. In contrast to attention, which focuses on the relevant parts of the image, Inverted Attention [45] proposed by Huang et al., inverts the attention and focuses on the complementary parts, resulting in diverse features. The attention is inverted along both spatial and channel dimensions.

## 3. Method

**Figure 3:**
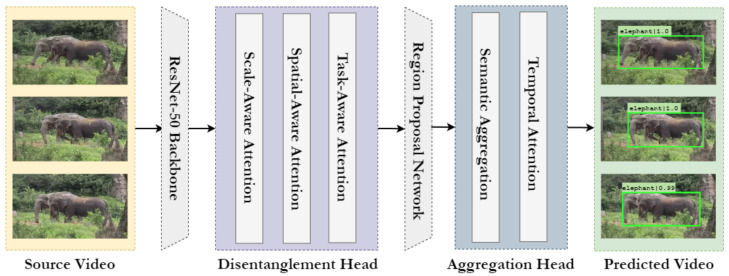
Overview of the proposed object detection framework based on the Faster R-CNN architecture. The figure highlights the modules with their components and illustrates the sequence in which the data is processed.

The proposed approach is a two-stage object detection framework based on the Faster R-CNN architecture. The object detection framework is implemented using openmmlab [46], an open-source toolbox based on PyTorch. An overview of the framework is presented in Figure 3, and the components are discussed further in the following sections.

### 3.1. Backbone

**Figure 4:**
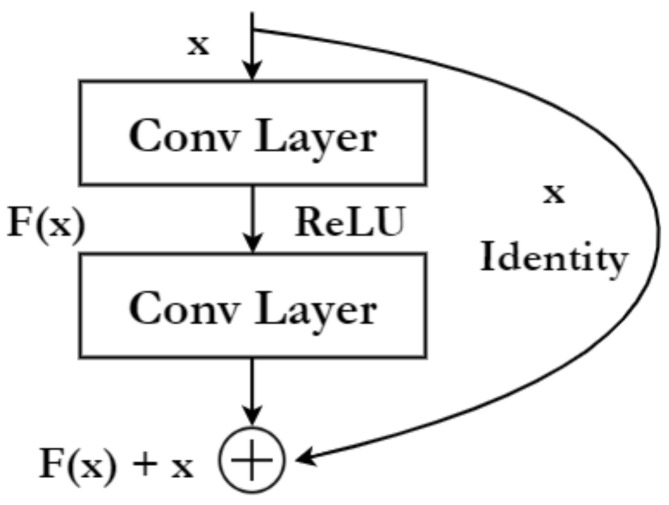
A single residual block in ResNet. Residual blocks are stacked together to form different variants of ResNet.

The backbone comprises a network that acts as a base feature extractor for object detection. It takes an image as the input and outputs the corresponding feature maps, which are then propagated to the subsequent modules for further processing. In this work, the backbone is implemented using ResNet [47]. Residual Neural Networks or ResNet are neural networks based on the idea of skip connections, which allows the skipping of one or more layers. Different variants of the ResNet are implemented by stacking these residual blocks; a residual block is shown in Figure 4. Skip connections solve the problem of vanishing gradients found in deep networks and also overcomes the degradation problem of accuracy saturation.

### 3.2. Disentangled Head

**Figure 5:**
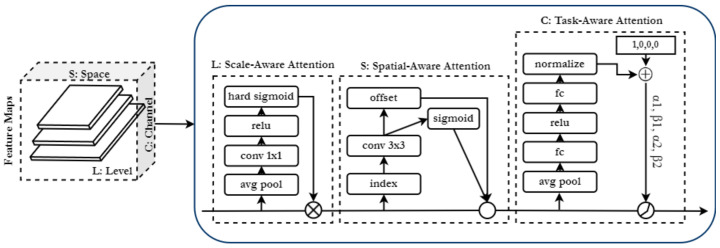
Overview of the neck implementing the disentanglement head. The individual attention mechanisms are enclosed within dashed boxes, and together, they form a single block represented by the solid box. The directed solid line indicates the sequence of processing, and the circles represent the functions applied within each attention. In task-aware attention, the initial values of [α1,β1,α2,β2] = [1,0,0,0] are concatenated with the normalised data and finding the maximum between the piecewise functions involving α1,β1,α2,β2 and the input tensor.

The backbone outputs feature maps at multiple levels, which are further processed by the disentanglement head. Figure 5 gives an overview of the disentanglement head, which is a sequence of repeated spatial, scale and task-aware attention mechanisms represented within dashed boxes. The solid directed line indicates the sequence of processing, and the circles represent the functions applied within an attention mechanism. In scale-aware attention, it is the output from the hard sigmoid multiplied by the tensor. In spatial-aware attention, it is deform_conv2d applied to offset and the sigmoid output. In task-aware attention, the initial values of [α1,β1,α2,β2] = [1,0,0,0] are concatenated with the normalised data and find the maximum between the piecewise functions involving α1,β1,α2,β2 and the tensor. The disentanglement head processes all the input video frames, to be later used as support frames and target frames in the aggregation head. Given a feature tensor from *L* different levels of a feature pyramid, $F\in R^{L\times S\times C}$, where S=H×W is the height and width and *C* is the number of channels. The general form of applying attention is shown in Equation (1).

(1) Attention=f(g(x),x)

where f(g(x),x) is the process of applying the generated attention g(x) to the input *x*. Applying the attention function across all dimensions would make it computationally expensive. Instead, the disentanglement network splits into three sequential attentions applied individually and independently, as shown in Equation (2), making it computationally efficient.

(2) $$W\left(F\right)=\pi_{C}\left(\pi_{S}\left(\pi_{L}\left(F\right).F\right).F\right).F$$

where $\pi_{L}$, $\pi_{S}$, and $\pi_{C}$ denote the scale, spatial and channel-wise attention modules, respectively, and *F* represents the spatial features of the frame generated by the backbone network.

- Scale-aware attention $\pi_{L}$ is utilised on the level dimension, emphasizes the varying scales of the objects and dynamically fuses the features based on their semantic importance.
- Spatial-aware attention is deployed on the space dimension *S* and emphasizes on the location of the objects, it is applied to the fused features from the scale aware-attention.
- Task-aware attention $\pi_{C}$ is applied on the channels; it exploits the feature channels by dynamically switching on and off to favour specific tasks.

### 3.3. Region Proposal Network

The Region Proposal Network (RPN) is a convolutional-based neural network that performs classification and regression. The classifier determines whether a proposal contains the object, whereas the regressor is used to determine the coordinates of the object in the proposal. Anchors of varying size and scale are first drawn over the input image; the RPN then determines whether the anchor contains an object. Varying thresholds are used to filter out anchors with low Intersection over Union (IoU); the remaining high-quality anchors are passed on to the subsequent stage for further processing.

### 3.4. Aggregation Head

**Figure 6:**
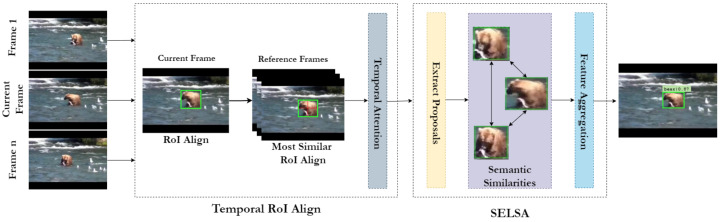
Overview of the aggregation head implemented using SELSA and Temporal RoI Align. The figure illustrates leveraging multiple frames as a reference for improving object detection.

The head is used for specific task outputs, such as bounding box or mask prediction. Here, we use Sequence-Level Semantics Aggregation (SELSA) [15] for video object detection as the Region of Interest (RoI) head and the Temporal RoI [16] align as the RoI extractor. An overview of the head is presented in Figure 6. As discussed previously, the goal of the SELSA module is to fully use features from all the frames, which would lead to more descriptive and robust features rather than just using the neighbouring frames. SELSA works by first extracting features from all the frames in the video, and then the semantic similarities of the features are calculated. Finally, features from the other frames are aggregated based on their similarities, resulting in improved object detection.

The traditional RoI align [48] utilizes only the current frame feature map for proposals. As a result, the features extracted do not contain temporal information. Temporal RoI align incorporates temporal information by using feature maps from other frames for the current frame proposals. First, the RoI features are extracted from the target frame feature map. The most similar RoI align module extracts the most similar RoI features from the multiple support frame feature maps depending on the K most similar points. Finally, the Temporal Attentional Feature Aggregation with N attention blocks performs temporal attention to produce the temporal RoI features. Further details regarding the working of Temporal RoI can be found in [16].

## 4. Experimental Setup

### 4.1. Dataset and Evaluation Metrics

In this section, we briefly discuss the datasets and the metrics we use for training and evaluating the proposed object detection framework. We use the ImageNet VID [49] from ImageNet Large Scale Visual Recognition Challenge 2015 (ILSVRC2015); it is the most commonly used dataset and benchmark for video object detection. The dataset consists of a subset of 30 classes from the object detection task. The training and validation sets contain 3862 and 555 videos, respectively. Additionally, we use still images from the overlapping classes of ImageNet DET data for training.

The performance of the object detection models is evaluated using Mean Average Precision (mAP). The calculation of the mAP, along with its associated concepts, are discussed below:

- Precision: Ratio of correctly predicted bounding boxes to all the predicted samples. It measures the model’s ability to identify only relevant objects.
  (3) $$Precision=\frac{\mathrm{Correctly}\;\mathrm{Predicted}\;\mathrm{Bounding}\;\mathrm{Boxes}}{\mathrm{All}\;\mathrm{Predicted}\;\mathrm{Bounding}\;\mathrm{Boxes}}$$
- Recall: Ratio of correctly predicted bounding boxes to all ground truth bounding boxes. It measures the prediction of all relevant cases.
  (4) $$Recall=\frac{\mathrm{Correctly}\;\mathrm{Predicted}\;\mathrm{Bounding}\;\mathrm{Boxes}}{\mathrm{Ground}\;\mathrm{Truth}\;\mathrm{Bounding}\;\mathrm{Boxes}}$$
- Intersection over Union (IoU): Measure of the overlap between the predicted bounding box and the ground truth. The IoU threshold is used to classify detection; only if the overlapping area is greater than a threshold t, the detection is regarded as correct.
  (5) $$IoU=\frac{\mathrm{Area}\;\mathrm{of}\;\mathrm{Intersection}\;\mathrm{between}\;\mathrm{Prediction}\;\mathrm{and}\;\mathrm{Ground}\;\mathrm{Truth}\;}{\mathrm{Area}\;\mathrm{of}\;\mathrm{Union}\;\mathrm{between}\;\mathrm{Prediction}\;\mathrm{and}\;\mathrm{Ground}\;\mathrm{Truth}}$$
- Average Precision (AP): Interprets the precision–recall curve as the weighted mean of precisions at each threshold, with the increase in recall from the preceding threshold used as the weight.
  (6) $$AP=\sum_{n}\left(R_{n}-R_{n-1}\right)P_{n}$$
- Mean Average Precision (mAP): Evaluates the accuracy of object detectors by averaging the average precision across all classes as shown below, where $AP_{i}$ is the average precision of $i^{th}$ class and *N* is the number of classes.
  (7) $$mAP=\frac{1}{N}\sum_{i=1}^{N}AP_{i}$$
  mAP is calculated at different IoU thresholds, mAP is the average mAP over 10 IoU thresholds from 0.5 to 0.95 with a step size of 0.05. mAP_50_ is the mAP at IoU = 0.5 and mAP_75_ is the mAP at IoU = 0.75.

### 4.2. Implementation Details

We experiment with ResNet-50 and ResNet-101 as the backbone network and carry out ablation studies and parameter optimisation using the smaller ResNet-50 model. The configured parameters are then used for training the ResNet-101 model. The backbone extracts the features from the frames, which are then processed by a sequence of attention blocks, and the output of the final task-attention mechanism is given to the region proposal network. The RPN consists of 12 anchors from 4 scales and 3 aspect ratios. The selected proposals are further processed by the Temporal RoI align operator, which replaces the traditional RoI align operator. The final classification and bounding box regression are implemented using SELSA. As part of the pipeline, the data is augmented through methods such as padding, flipping, resizing and normalisation. The backbone network is initialised with the ImageNet pre-trained weights. We train all the models with identical configurations, and each model is trained for seven epochs. The models were trained using 4 NVIDIA A100 GPUs with a batch size of 4 and SGD as the optimizer. The learning rate is initially set to 0.005 and is stepped down by 10 at the fourth and sixth epochs.

## 5. Experiments and Results

### 5.1. Ablation Study

In this section, we evaluate and discuss the performance of the proposed object detection framework using different backbones. First, we conduct several ablation studies by varying different parameters to determine the optimal configuration. All the other constraints were constant, and ResNet-50 was used as the backbone. The best results are highlighted in bold.

#### 5.1.1. Number of Attention Blocks

An attention block consists of scale, spatial and task-aware attention mechanisms. The depth of the attention head can be increased by stacking the attention blocks in conjunction. We train the effect of varying the number of attention blocks, and the results are presented in Table 1. We observe that increasing the number of blocks decreases the performance, and we achieve the best results using two blocks. This decrease in performance can be attributed to overfitting the data, particularly with the ImageNet VID dataset, which has only a few objects per frame.

#### 5.1.2. Number of Most Similar ROI Points

The most similar ROI align extracts the most similar ROI features from the support frame feature maps for the target frame proposals. A similarity map for each support frame is computed, and the top K most similar points are projected. Using these points, the most similar ROI features are extracted from the support frame feature maps. The effect of the number of similar points is presented in Table 2.

#### 5.1.3. Number of Temporal Attention Blocks

The temporal attention block is used for aggregating the features extracted from the target frame feature map and support frames feature maps. The input in the form of ROI features is split into N groups for N temporal attention blocks, and each block generates an attention map. The output across all the temporal attention blocks is concatenated to obtain the temporal ROI features. The performance of varying the temporal attention is presented in Table 3.

#### 5.1.4. Effectiveness of the Disentanglement Attention Module

We evaluate the performance of the proposed framework against the corresponding SELSA and Temporal RoI models as the baseline. The results using ResNet-50 are presented in Table 4, and using ResNet-101 in Table 5. We observe an improvement in the performance with the addition of the disentanglement head against both SELSA and TROI baselines.

### 5.2. Main Results

In Table 6, we compare the model against the state-of-the-art models on the ImageNet VID dataset. From the table, we can observe that the proposed framework outperforms the existing methods on the ResNet-50 backbone by achieving a new best mAP of 80.2%. With ResNet-101, we improve the performance (mAP) over the baseline model SELSA by 2.15 and TROI by 0.4 points. It is important to mention that our proposed disentanglement head is a plug-and-play module and can be integrated into any video object detection method to improve performance. The results summarized in Table 6 are achieved when our module is crafted in TROI [16]. However, we argue that incorporating our module into enhanced methods will bring significant performance gains and produce state-of-the-art results.

### 5.3. Qualitative Analysis

#### 5.3.1. Visualising Detection Results

**Figure 7:**
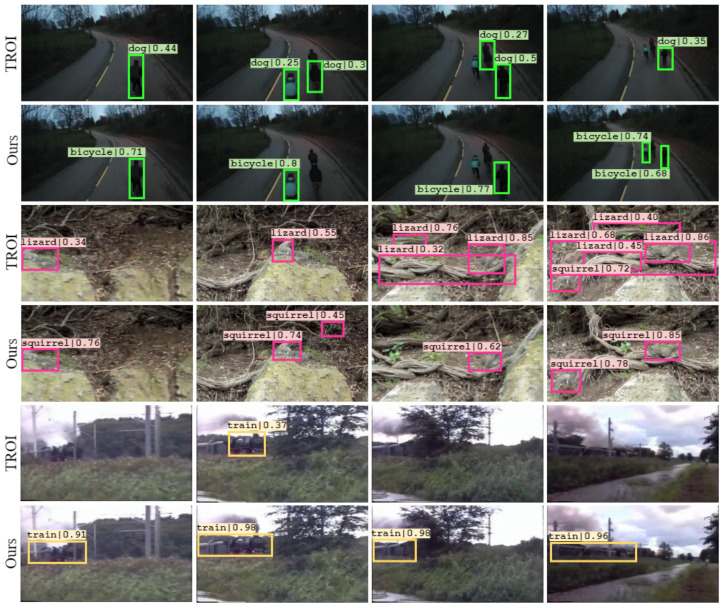
Visualising the performance of the proposed model against the baseline on the ImageNet VID dataset. Our model performs better in challenging conditions with fewer misclassifications and false positives.

The result from detection is visualised and compared in Figure 7. From the figure, we can observe that our model performs better in comparison with the baseline under difficult conditions. This can be attributed to the inclusion of an attentional head, which improves the detection of objects of varying scales and sizes. In the first two cases, the baseline model misclassifies the object, whereas our model correctly identifies the object. This misclassification becomes critical and detrimental as it is propagated across subsequent frames. In the last case, the moving train is observed at different scales and viewpoints, which is effectively processed and detected using scale and spatial attention.

#### 5.3.2. Robustness against Challenges in VOD

**Figure 8:**
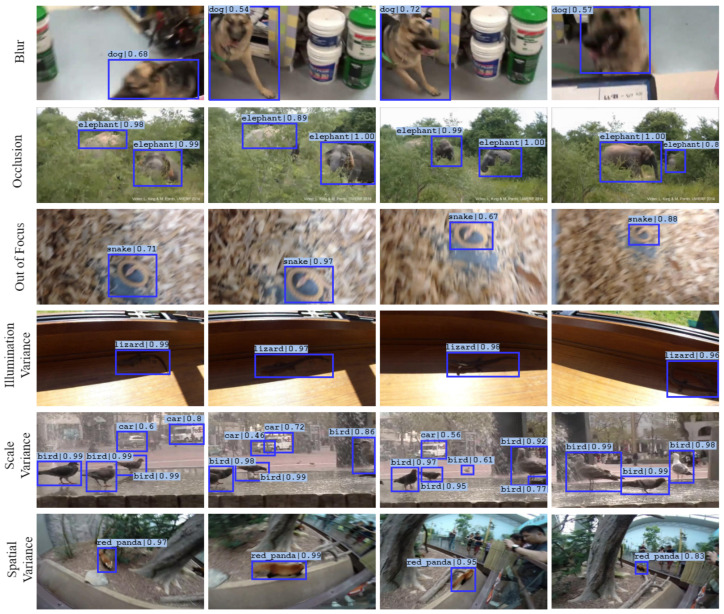
Video object detection under challenging conditions. The figure demonstrates the robustness of the proposed approach against inherent challenges in videos.

We also emphasize and present the performance of the model under challenging conditions of video object detection discussed previously. From Figure 8, we can discern the robustness of the model towards these challenges. As discussed previously, we tackle the challenges of blur, occlusion, out-of-focus and illumination variance by leveraging the temporal information across the frames. The challenges of variance in scale and spatial locations, which were observed in the baseline, are overcome through the proposed model.

#### 5.3.3. Failure Case Analysis

**Figure 9:**
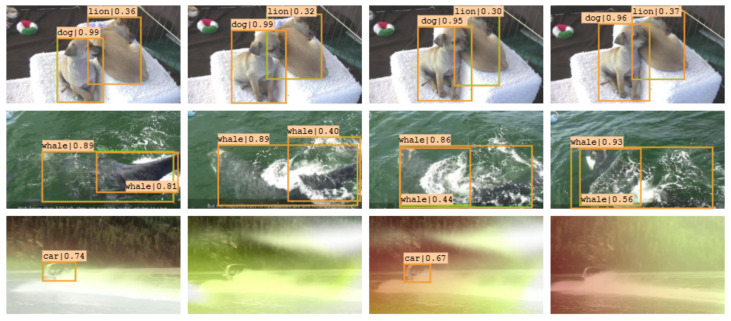
Fail cases of the proposed model: misclassified objects (top), duplicate bounding boxes (middle) and inaccurate or missing predictions (bottom).

In Figure 9, we present some of the cases where the model has failed to detect the objects correctly. In the first row, we can observe that the model consistently misclassifies an object throughout the video. The dog instance in the foreground is identified accurately. In the other instance, where discriminative features such as the snout are hidden, it is misclassified as a lion with low confidence. This misclassification can also be attributed to the colour of the dog’s coat, which is similar to that of a lion. Next, we have cases where the model predicts multiple bounding boxes for a single object. The duplicate bounding boxes are suppressed using Non-Maximum Suppression (NMS), a post-processing technique. NMS filters the predicted bounding boxes based on confidence and IoU thresholds. These thresholds are static parameters, and the challenge lies in selecting thresholds suitable across all cases. A high threshold may result in valid predictions being discarded, whereas a low threshold results in several predictions with lower confidence and overlap. Addressing this shortcoming associated with greedy NMS, Zhou et al [59]. propose Nearby Objects Hallucinator (NOH), which uses a Gaussian distribution to detect other objects near a proposal. Combined with NMS, the NOH-NMS solves the rigid NMS threshold problem by being aware of nearby objects during suppression. At the bottom, we have cases of temporally inconsistent predictions where the model misclassifies or fails to detect the object. We can see this occurs in cases of extremely deteriorated frames. Due to the deterioration, the model cannot determine the most similar points across frames for leveraging temporal information. This can be considered an example of object detection under challenging conditions. Ahmed et al [12] discuss these challenges and propose directions for alleviating them.

## 6. Conclusions

In this work, we first raise an important problem of naive attention-based feature aggregation that hinders the upper bound in existing state-of-the-art video object detection methods. To mitigate this challenging problem, motivated by [18], we disentangle the feature representation generated from the backbone network into scale, spatial and task-wise features prior to feature aggregation. Upon integrating our disentanglement head prior to feature aggregation in recent video object detection methods, we observe consistent and significant performance improvements. When our module is adopted into the recent state-of-the-art method TROI [16], we achieve a new state-of-the-art mAP of 80.3% on the ResNet-50 backbone network. The increase over the baseline models ascertains the contributions of disentangling features in improving video object detection. Moreover, the proposed method can be effortlessly integrated into any video object detection method to improve performance. We hope this work inspires future researchers to focus on developing approaches that learn more discriminative features besides feature aggregation methods in video object detection.

## Figures and Tables

**Table 1:** Comparison of performance with varying number of Dynamic Head blocks.

Blocks	mAP	mAP_50_	mAP_75_
1	0.484	0.780	0.515
**2**	**0.495**	**0.797**	**0.537**
3	0.485	0.771	0.521
4	0.485	0.770	0.518
5	0.477	0.756	0.509
6	0.451	0.732	0.476

**Table 2:** Comparison of performance with varying numbers of most similar RoI points.

Points	mAP	mAP_50_	mAP_75_
1	0.492	0.794	0.531
2	0.496	**0.802**	0.537
3	0.492	0.789	0.527
4	0.493	0.800	0.525
5	0.495	0.795	0.532
**6**	**0.498**	0.799	**0.540**

**Table 3:** Comparison of performance with varying number of Temporal Attention blocks.

Blocks	mAP	mAP_50_	mAP_75_
**1**	**0.497**	0.797	**0.544**
2	0.494	0.791	0.538
4	0.493	**0.800**	0.525
8	0.487	0.781	0.522
16	0.489	0.775	0.518
32	0.484	0.782	0.518

**Table 4:** Comparison of the proposed framework against the baseline using ResNet-50.

Method	mAP	mAP_50_	mAP_75_
SELSA	0.487	0.784	0.531
SELSA + Disentanglement Head	0.490	0.788	0.536
TROI	0.485	0.798	0.523
TROI + Disentanglement Head	**0.498**	**0.802**	**0.544**

**Table 5:** Comparison of the proposed framework against the baseline using ResNet-101.

Method	mAP	mAP_50_	mAP_75_
SELSA	0.524	0.802	0.579
SELSA + Disentanglement Head	0.523	0.816	0.589
TROI	0.516	0.820	0.563
TROI + Disentanglement Head	**0.525**	**0.824**	**0.577**

**Table 6:** Comparison of the proposed framework against recent state-of-the-art methods on the validation set of the ImageNet VID benchmark.

Method	Detector	Backbone	mAP
DFF [27]	R-FCN	ResNet-50	70.3
FGFA [28]	R-FCN	ResNet-50	74.0
D&T [36]	R-FCN	ResNet-50	76.5
RDN [50]	Faster R-CNN	ResNet-101	76.7
MEGA [33]	Faster R-CNN	ResNet-50	77.3
SELSA [15]	Faster R-CNN	ResNet-50	78.4
TROI [16]	Faster R-CNN	ResNet-50	79.8
**Ours**	Faster R-CNN	ResNet-50	80.2
Impression Net [51]	R-FCN	ResNet-101	74.2
FGFA [28]	R-FCN	ResNet-101	76.3
LSTS [52]	Faster R-CNN	ResNet-101	77.2
MA-Net [53]	R-FCN	ResNet-101	78.1
THP [54]	R-FCN	ResNet-101	78.6
STSN [55]	R-FCN	ResNet-101	78.9
OGEMN [56]	R-FCN	ResNet-101	79.3
D&T [36]	R-FCN	ResNet-101	79.8
PSLA [34]	R-FCN	ResNet-101	80.0
SELSA [15]	Faster R-CNN	ResNet-101	80.25
STMN [31]	R-FCN	ResNet-101	80.5
LRT-RN [57]	Faster R-CNN	ResNet-101	81.0
RDN [50]	Faster R-CNN	ResNet-101	81.8
TROI [16]	Faster R-CNN	ResNet-101	82
HVRNet [58]	Faster R-CNN	ResNet-101	83.2
**Ours**	Faster R-CNN	ResNet-101	82.4

## Data Availability

Not applicable.
